# Machine Learning-Based Analysis of Digital Movement Assessment and ExerGame Scores for Parkinson's Disease Severity Estimation

**DOI:** 10.3389/fpsyg.2022.857249

**Published:** 2022-03-17

**Authors:** Dunia J. Mahboobeh, Sofia B. Dias, Ahsan H. Khandoker, Leontios J. Hadjileontiadis

**Affiliations:** ^1^Department of Electrical Engineering and Computer Science, Khalifa University, Abu Dhabi, United Arab Emirates; ^2^CIPER, Faculdade de Motricidade Humana, Universidade de Lisboa, Lisbon, Portugal; ^3^Department of Biomedical Engineering, Khalifa University, Abu Dhabi, United Arab Emirates; ^4^Healthcare Engineering Innovation Center (HEIC), Khalifa University, Abu Dhabi, United Arab Emirates; ^5^Department of Electrical and Computer Engineering, Aristotle University of Thessaloniki, Thessaloniki, Greece

**Keywords:** Parkinson's Disease (PD), Personalized Serious Game Suite (PGS), intelligent Motor Assessment Tests (iMAT), machine learning (KNN SVM RF), PD staging, i-PROGNOSIS

## Abstract

Neurodegenerative Parkinson's Disease (PD) is one of the common incurable diseases among the elderly. Clinical assessments are characterized as standardized means for PD diagnosis. However, relying on medical evaluation of a patient's status can be subjective to physicians' experience, making the assessment process susceptible to human errors. The use of ICT-based tools for capturing the status of patients with PD can provide more objective and quantitative metrics. In this vein, the Personalized Serious Game Suite (PGS) and intelligent Motor Assessment Tests (iMAT), produced within the i-PROGNOSIS European project (www.i-prognosis.eu), are explored in the current study. More specifically, data from 27 patients with PD at Stage 1 (9) and Stage 3 (18) produced from their interaction with PGS/iMAT are analyzed. Five feature vector (FV) scenarios are set, including features from PGS or iMAT scores or their combination, after also taking into consideration the age of patients with PD. These FVs are fed into three machine learning classifiers, i.e., K-Nearest Neighbor (KNN), Support Vector Machines (SVM), and Random Forest (RF), to infer the stage of each patient with PD. A Leave-One-Out Cross-Validation (LOOCV) method is adopted for testing the classification performance. The experimental results show that a high (>90%) classification accuracy is achieved from both data sources (PGS/iMAT), justifying the effectiveness of PGS/iMAT to efficiently reflect the motor skill status of patients with PD and further potentiating PGS/iMAT enhancement with a machine learning a part to infer for the stage of patients with PD. Clearly, this integrated approach provides new opportunities for remote monitoring of the stage of patients with PD, contributing to a more efficient organization and set up of personalized interventions.

## 1. Introduction

Parkinson's Disease (PD) is one of the most frequent neurodegenerative diseases affecting approximately 2% of the population aging 65 years and older, whereas patients of 85 years accumulated 4% of the population (Opara et al., 2017). The progressive neurological disorder is distinguished by rigidity and tremors causing patients to exert slowness in movement, postural instability, and other chronic symptoms (Blochberger and Jones, 2011). Motor limitations are usually gathered by clinicians as part of the motor assessment process for the sole purpose of treatment decisions reflecting the patient's overall disability in a standardized measurement tool, such as the Movement Disorder Society-Unified Parkinson's Disease Rating Scale (MDS-UPDRS Part III) (Martinez and Forjaz, 2006; Goetz et al., 2008). However, clinicians' judgments can be subjective to their experience affecting the assessment of the PD severity. Nevertheless, an alternative measuring system can be reasonably accepted which exhibits a high correlation with the common globally used standard rating scales, MDS-UPDRS Part III subscores, for monitoring the progression of PD in patients (Yang et al., 2016; Dias et al., 2020b). This actively demonstrates that the healthcare domain has adopted several body-tracking systems like the Microsoft Kinect^®^ sensor (Knippenberg et al., 2017), widely used in neurological rehabilitation programs for clinical measurements of motor functions for monitoring patients with PD, assessing their gait, body balance, hand tracking, and analyzing their posture and limb tasks (Ferraris et al., 2014; Galna et al., 2014; Stone and Skubic, 2014; Yang et al., 2014; Rocha et al., 2015). Furthermore, the MentorAge^®^ sensor emerged as an alternative tracking system to the Kinect device manifesting high competence in real-life scenarios (Anzivino et al., 2018; Petsani et al., 2019).

With the availability of new Natural User Interfaces (NUIs), combined with the concept of serious games, efforts were placed in creating personalized serious games (SGs), such as the PGS Dias et al. (2020c) from the i-PROGNOSIS project (www.i-prognosis.eu), as both intervention and/or motor assessment tool for monitoring and tracking the behavioral change of patients with PD in terms of gait, agility, balance, and coordination impairments, as well as assess patients in adhering to physical therapy through enrolling in a gamified environment (Anzivino et al., 2018). Moreover, one of the deployed intelligent tools that can be seen as an alternative solution for the standard rating scales, MDS-UPDRS Part III subscores, is the intelligent Motor Assessment Tests (iMAT) (Dias et al., 2020b) from the i-PROGNOSIS project. Although the diagnosis of PD commonly relies on motor symptoms, non-motor symptoms such as cognitive changes related to attention and other conditions have been studied as supportive means for early diagnosis (Postuma et al., 2015). A revolutionary substitute by several researchers studied the application of machine learning as measures for PD diagnosis and assessment with the help of analyzing motor symptoms, kinematics, and wearable sensor data (Ahlrichs and Lawo, 2013; Ramdhani et al., 2018; Belić et al., 2019).

Based on the aforementioned, the present study extends the study presented in Dias et al. (2020b), by identifying the potentiality to correctly estimate the severity of the PD, as measured by clinical metrics, *via* machine learning analysis of the iMAT and PGS scores. In this endeavor, the data reported in Dias et al. (2020b), drawn from 27 patients with early PD, are further analyzed under various testing scenarios employing machine learning, leading to encouraging results regarding the potentiality to combine iMAT with PGS, in order to efficiently estimate the severity of patients with PD.

The present study is structured as follows: In Section 2, a related background on the main topics addressing PD rehabilitation, SGs, and i-PROGNOSIS PGS/iMAT characteristics is presented. In Section 3, the dataset characteristics along with the proposed methodology are provided. Furthermore, Section 4 highlights and discusses the results and the efficiency of the proposed approach along with the related limitations, including future extensions. Finally, Section 5, concludes the article.

## 2. Background

### 2.1. PD Rehabilitation and SGs

Multiple pieces of evidence revealed that patients with PD suffer from progressive deterioration in their disability, despite following the recommended medical prescriptions (Ellis et al., 2008; Abbruzzese et al., 2016). Exercise and physical activity were found to be directly correlated with enhancing the health and well-being of patients with PD. Rehabilitation therapies for PD primarily focus on improving patients' overall quality of life, maximizing their level of mobility and activity (Soh et al., 2012). In spite of several proves of adopting physical activities in rehabilitation programs is powerful means for reducing the risk of causing PD, the current conventional programs have been discovered to be heterogeneous, sub-optimal, and lack common consensus (Abbruzzese et al., 2016). In addition, supporting exercise-based rehabilitation programs continues to face challenges in cost, accessibility, patient adherence, and acceptability (Barry et al., 2014). Abbruzzese et al. (2016), proposed an innovative substitute by introducing therapeutic techniques, such as Motor Imagery (MI) and Action Observation Therapy (AOT) (Abbruzzese et al., 2016). Through matching the internal representations with the imagined and observed actions from these methods, patients' motor skills are enhanced and new learning tasks are built. Nevertheless, some practical limitations occur with its adoption as an intervention for PD rehabilitation (Abbruzzese et al., 2016). Knippenberg et al. (2017), considered the use of Motion Capture Systems (MCS), such as the Microsoft Kinect^®^ sensor, for patients with neurological disorders like PD. Assessment results from its use at rehabilitation centers showed its potential to assess patients in body balance and fall prevention (Stone and Skubic, 2014; Yang et al., 2014), during their clinical measurement of motor functions and gait assessments (Galna et al., 2014; Rocha et al., 2015), body balance, hand tracking, and analyzing their posture and limb tasks (Ferraris et al., 2014, 2019; Rocha et al., 2015; Otte et al., 2016; Yang et al., 2016). Additionally, the MentorAge^®^ sensor emerged as an alternative tracking system to the Kinect device, manifesting high competence and proven to be a better alternative for its potentiality in real-life scenarios (Anzivino et al., 2018; Petsani et al., 2019).

Over the years, it has been highlighted that SGs are being adopted in the healthcare domain, as they benefit patients in the physical, mental, and social well-being aspects. SGs are digital games that provide players not only entertainment but (re)educational value as well (Caserman et al., 2020). Such health interventions are noticed to enhance old patients' health, as well as, increase patients' adherence in general. The main benefit of adopting SGs as health interventions is that they act as incentives for patients to enhance their performance and challenge themselves to continuous improvement on higher serious game levels. Besides, they assist doctors and other healthcare providers in personalizing a plan for each patient, based on their performance using the SGs as health interventions. Nonetheless, digital SGs allow on-site data capturing, which contributes to the collection of data-in-the-wild, representing the intuitiveness of the user's interactions with the SGs.

In fact, the collection of on-site data from the SGs, using the NUIs of Kinect^®^ or MentorAge^®^ sensors, corresponds to the term “in-game metrics”. The latter is an efficient and comprehensive research tool that boosts the effectiveness of the SGs, as they capture the changes in the physical and cognitive health state of the user. The in-game metrics are collected during the interaction of patients with a gamified platform. Also, for diagnostic processes, SGs can be administered in an unobtrusive ecologically proper environment in an Exercise Game (ExerGame) fashion (Barry et al., 2014; Konstantinidis et al., 2014). Several researchers investigated the use of metrics for the purpose of early detection and post-tests predictions. Alonso-Fernández et al. (2020), studied the collected in-game metrics of users from the gameplay and deployed it with machine learning algorithms to predict patients' outcomes while playing (Alonso-Fernández et al., 2020). Aguilar et al. (2017), estimated the probability of 60 years old participants' body movement, recorded by Kinect, using classification algorithms along with generalized linear models, to distinguish participants' group age. Pirovano et al. (2014), developed a solution that provides real-time feedback alarms during exercise using combined fuzzy-based monitoring and in-game adaptation. Nonetheless, in a gamified environment, ExerGames assess patients' physical health (Staiano and Calvert, 2011), and the respective derived data provide objective information to various parties of interest. The importance of ExerGames for older adults, and especially the ones with PD, were explored in the studies of Dias et al. (2020a,b,c, 2021).

### 2.2. The i-PROGNOSIS PGS/iMAT

#### 2.2.1. PGS Characteristics

**Figure 1:**
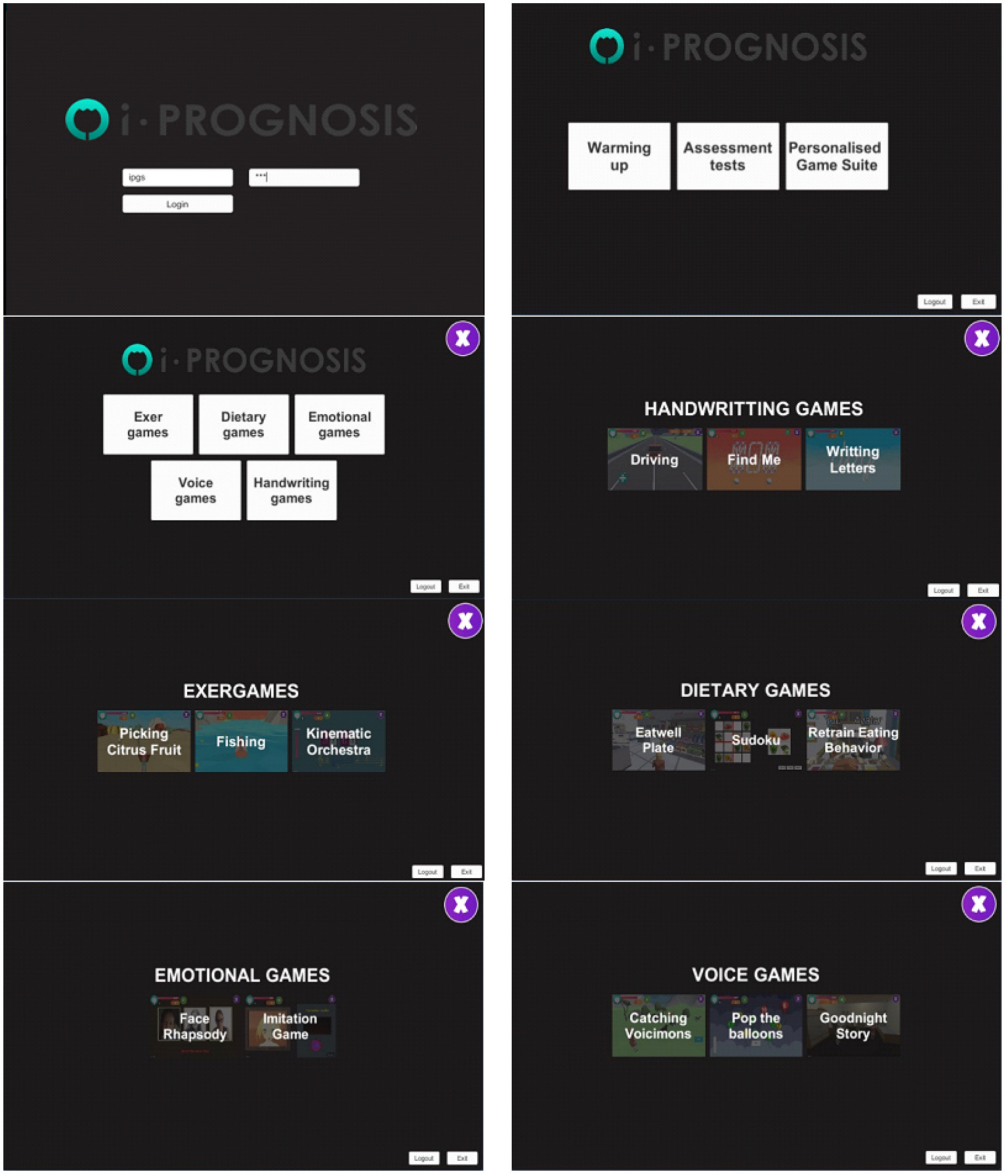
The overview of the i-PROGNOSIS PGS environment.

**Figure 2:**
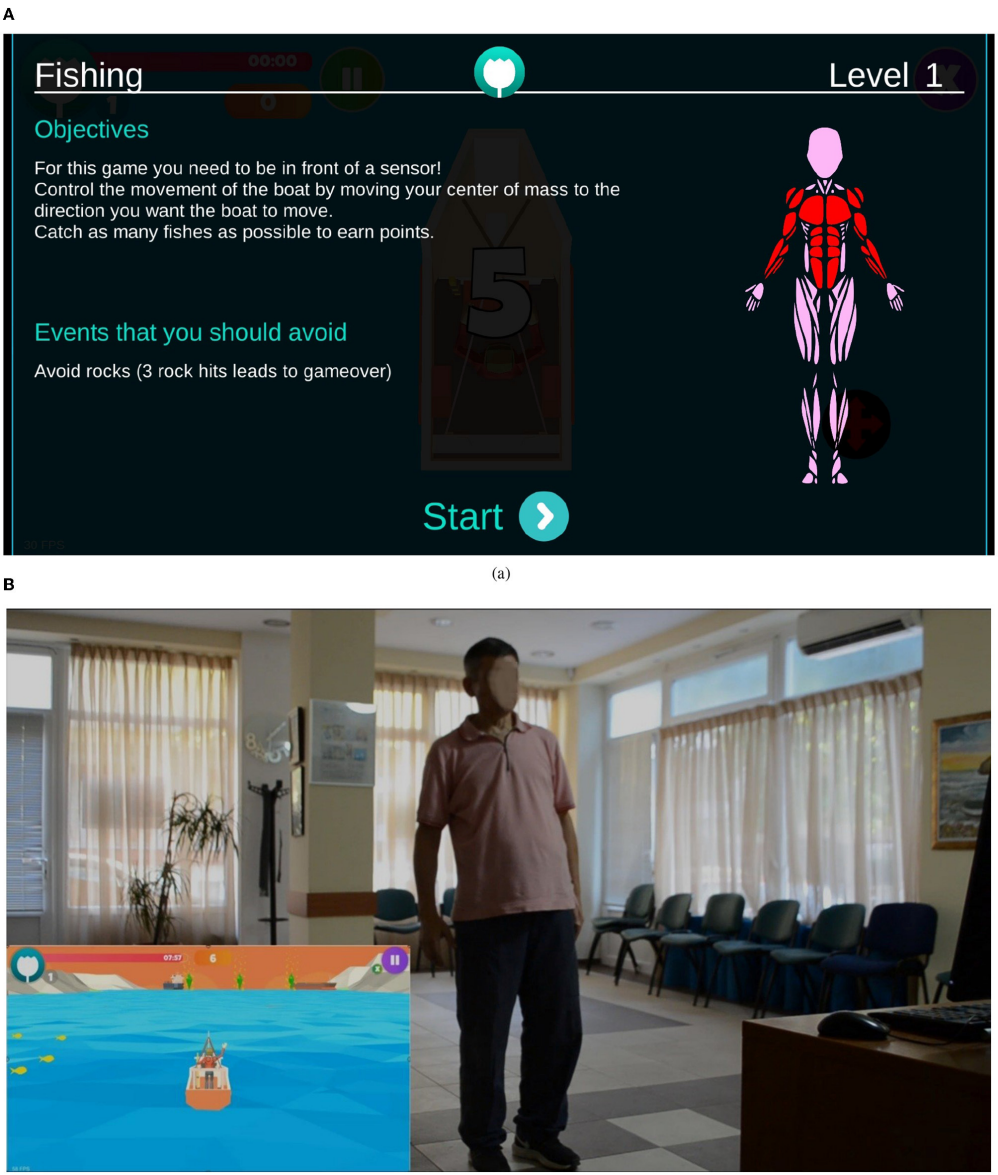
Excerpt from the i-PROGNOSIS PGS ExerGame “Fishing Game.” **(A)** The interface of the game, including the instructional information and visualization of the targeted muscle groups. **(B)** The game environment shows a patient with PD controlling the boat movement with his body.

The adopted i-PROGNOSIS PGS platform consists of PD-related SGs (Dias et al., 2021) that refer to exercise (“ExerGames,”) diet (“DietaryGames,”) emotions (“EmoGames,”) and voice (“VoiceGames”) (Figure 1). These SGs are co-designed by various stakeholders that relate to PD (e.g., patients with PD/older adults, physicians, carers, technology developers, and healthcare policy makers), in order to holistically address the needs of patients with PD and assist them in coping with the PD symptoms in their everyday living. The proposed study draws information from the use of the PGS ExerGames, and especially the “Fishing Game” (Figure 2) that mostly targets the training of the upper body muscles (Figure 2A). Overall, the objective of the “Fishing Game” is to catch as many fish as possible to earn points using the body center of mass as a controlling factor of the boat movement and direction (Figure 2B). In this way, patients with PD train their upper body muscles and increase or sustain their balance skills, in a gamified way.

The patients' interaction with the PGS is captured by the Nively MentorAge^®^, a depth RGB image sensor that operates on the Android system and uses an infrared 3D capturing technology, where it can detect a maximum of four people in a single room within a range of 0.6–5 m. The MentorAge^®^ is the controller that targets the patient's physical motion and provides the interface for capturing the body posture and gestures during their interaction with the platform through extracting and analyzing the body's skeleton and silhouette (http://www.nively.com/). Monitoring and sensing the patient's status provide useful feedback information expressed *via* the collected data. Furthermore, the anonymization of the collected data from the PGS platform is essential for preserving the privacy of the patients. The data that can be inputted and retrieved *via* the PGS are listed below:

1. Contact data and account credentials of patients and physicians.
2. Historical data taken from the game scenarios played by the patients in the personalized program, are labeled as: {Patient ID, Date, Game ID, Game Name, Level, Score, Skipped trials, Achieved goals, Time to achieve goals, and Accuracy}.
3. Statistical data presenting the patients' game score, goal achievements, and engagement level.
4. Game friends' activity data including achievements of the patient's connected game friends.
5. Medical history data of the patients and the results of their clinical assessment tests.

#### 2.2.2. iMAT Characteristics

**Figure 3:**
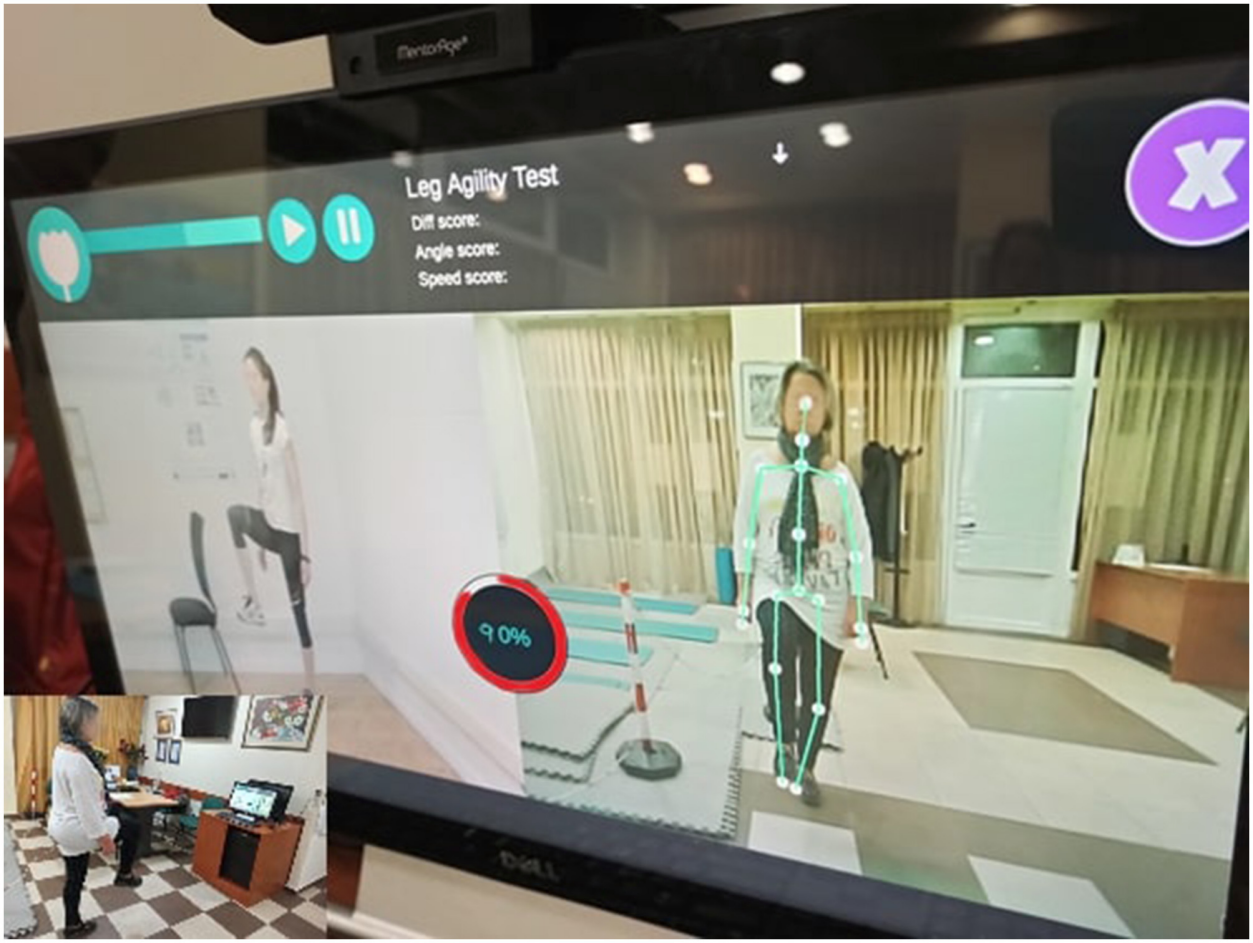
The i-PROGNOSIS iMAT assessment environment during its use by a patient with PD (right panel), trying to imitate the expert's leg posture (left panel), achieving high accuracy of 90%.

i-PROGNOSIS iMAT is a set of motor assessment tests (Dias et al., 2020b) that could be seen as a “digital twin” version of some conventional tests used in the clinical environment for motor skills assessment of patients with PD. In particular, the iMAT design was co-created by neurologists specializing in PD from Greece, UK, and Germany. Six tests are included (i.e., Test 1-Test 6), designed and developed following the MDS-UPDRS Part III examination that places the focus of assessment on postural, balance, agility, coordination, and hand movements. These are referenced as MDS-UPDRS Part III Items 18, 23, 24, 25, 26, and 28. Similar to the PGS, iMAT incorporates the data capturing interfacing of Nively MentorAge^®^, and, thus, it can be combined with the use of PGS. An example of the iMAT use in practice is depicted in Figure 3. As it can be seen from the latter, the patient with PD (right panel), while being at the premise, tries to imitate the leg movement of the expert (left panel) during Assessment Test 2 which examines the leg agility movements. The identified skeleton is overlaid on the video of a patient with PD and an accuracy percentage (here 90%) is provided as real-time feedback, motivating improvement toward 100% accuracy. Clearly, the use of iMAT does not require any visit to the clinical environment, and the accuracy per each test session is saved and transmitted to the attending physician *via* the i-PROGNOSIS Azure-based dashboard. More details about the iMAT could be found in Dias et al. (2020b).

## 3. Materials and Methods

### 3.1. Dataset

#### 3.1.1. Data Characteristics

For the proposed analysis, data drawn from patients with PD interacting with the “Fishing Game” and iMAT are used. These data corresponded to the clinical motor assessment data of patients with PD, performed by neurologists from the three different European medical centers (i.e., Greece, Germany, and the UK). The main characteristics embedded in the clinical dataset correspond to patients' username, ID, demographics, whether on medication or not, Hoehn and Yahr (H&Y) clinical rating scale, and their score in MDS-UPDRS Part III Items 18, 23, 24, 25, 26, and 28. The H&Y is a standard clinical rating scale that stages the functional disability associated with PD. The original H&Y scale consists of five stages, where Stage 1 has unilateral involvement, Stage 2 bilateral without impairment of balance, Stage 3 bilateral with impairment of posture, Stage 4 represents the severity of the disease associated with lack of physical independence, and finally, Stage 5 is for patients bounded by a bed or wheelchair (Goetz et al., 2004). However, based on the present dataset, the H&Y stages in this study are limited to one and three, labeled here as class 1 (Stage 1) and class 2 (Stage 3), respectively.

#### 3.1.2. Participants' Demographics

The dataset of the present study involved a total of 27 PD patients with a distribution of class1/class2 as 9/18. Patients undergoing this study ranged between the age of 43 and 79 years, with 69 years old as the highest number of patients and 62 years as the mean age. The patients in this study enrolled from three different countries, i.e., Greece, Germany, and the UK. The gender distribution was a ratio of 1 to 2, having 9 women and 18 men patients with PD, where the majority were male patients with PD. All patients were under PD medication except one participant. The data acquisition took place from September 2019 to January 2020, during periodic sessions (1 per month) of the iMAT within a controlled environment at the three medical centers. Each patient with PD performed each Assessment Test up to four times per session. Moreover, periodic sessions of the “Fishing Game” were also combined. It should be noted, however, that 21 (6/15) out of 27 patients with PD have provided combined scores from both PGS and iMAT. More details about the patients' selection process can be found in Dias et al. (2020b).

### 3.2. Proposed Analysis

**Figure 4:**
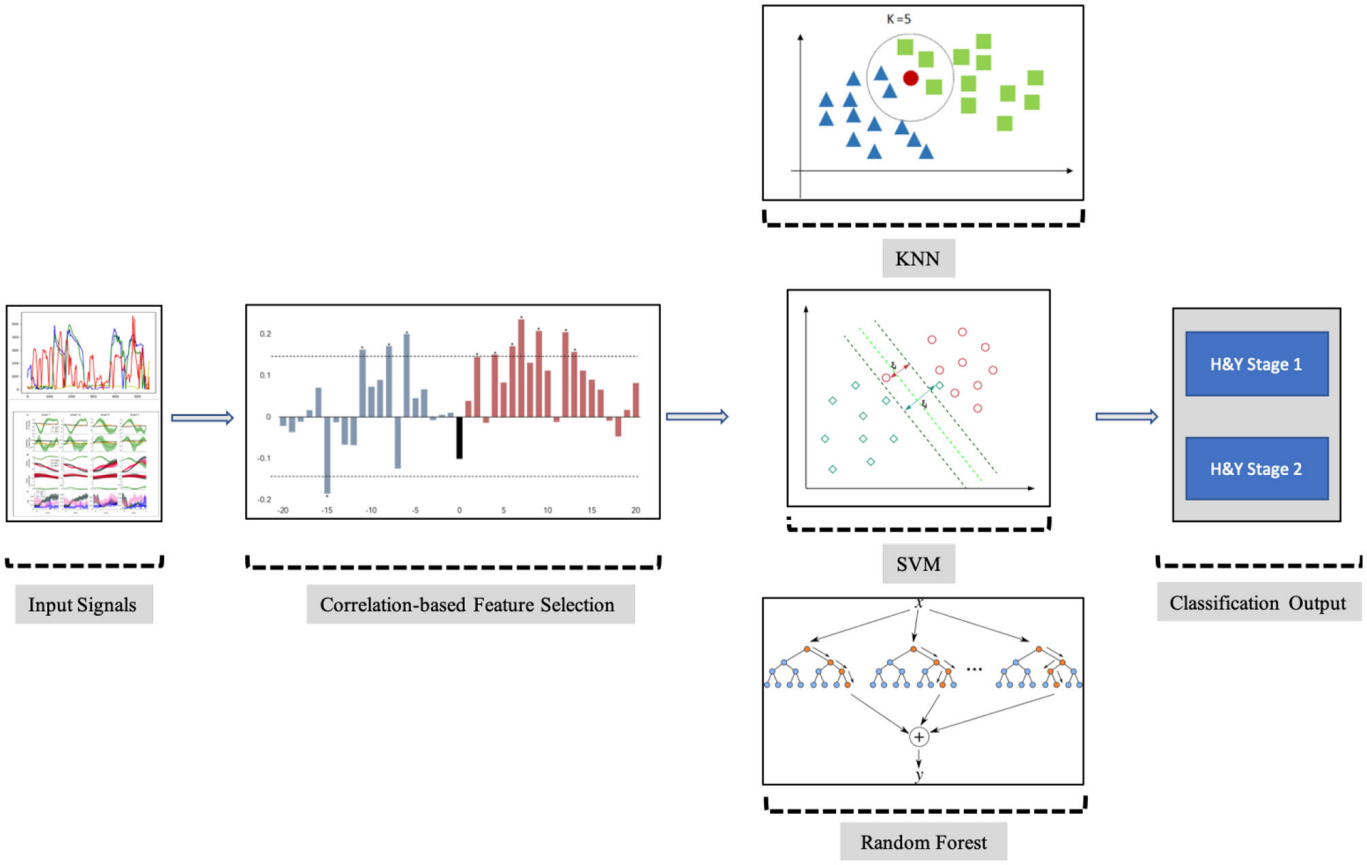
Block diagram of the steps employed in the proposed analysis.

The objective of the proposed analysis is to find and use the appropriate features from the PGS and iMAT scores that could efficiently classify the patients with PD into H&Y stage 1 or stage 2 of PD. This potentiates the use of PGS/iMAT as a means to monitor the symptom status of patients with PD in a non-clinical setting and capture any deterioration; hence, their possible transition from a lower to higher H&Y stage. The steps of the proposed analysis are schematically depicted in Figure 4 and described below.

#### 3.2.1. Feature Vector (FV) Selection

In order to form the FV that could provide the best classification output, various characteristics have been estimated from the captured data. In particular, the statistical parameters of average (avg), median (med), standard deviation (std), minimum (min), and maximum (max) values of the “Fishing Game” scores were used as features related to the PGS. In addition, based on the findings from Dias et al. (2020b), the median value of the iMAT test scores, i.e., *Timed, i* = 1, 2, …, 6, provided with the highest (absolute) correlation with the PD clinical scores when compared to the corresponding avg, std, min, and max values. Hence, the *Timed, i* = 1, 2, …, 6 values were used here as features related to the iMAT. Moreover, the participants' demographics of age and gender were also considered as features. In this vein, the following versions of the FV were initially adopted:

1. iMAT median scores,
2. iMAT median scores + demographics,
3. Statistical parameters of the “Fishing Game” score,
4. Statistical parameters of the “Fishing Game” score + demographics,
5. iMAT median scores + Statistical parameters of the “Fishing Game” score,
6. iMAT median scores + Statistical parameters of the “Fishing Game” score + demographics.

**Figure 5:**
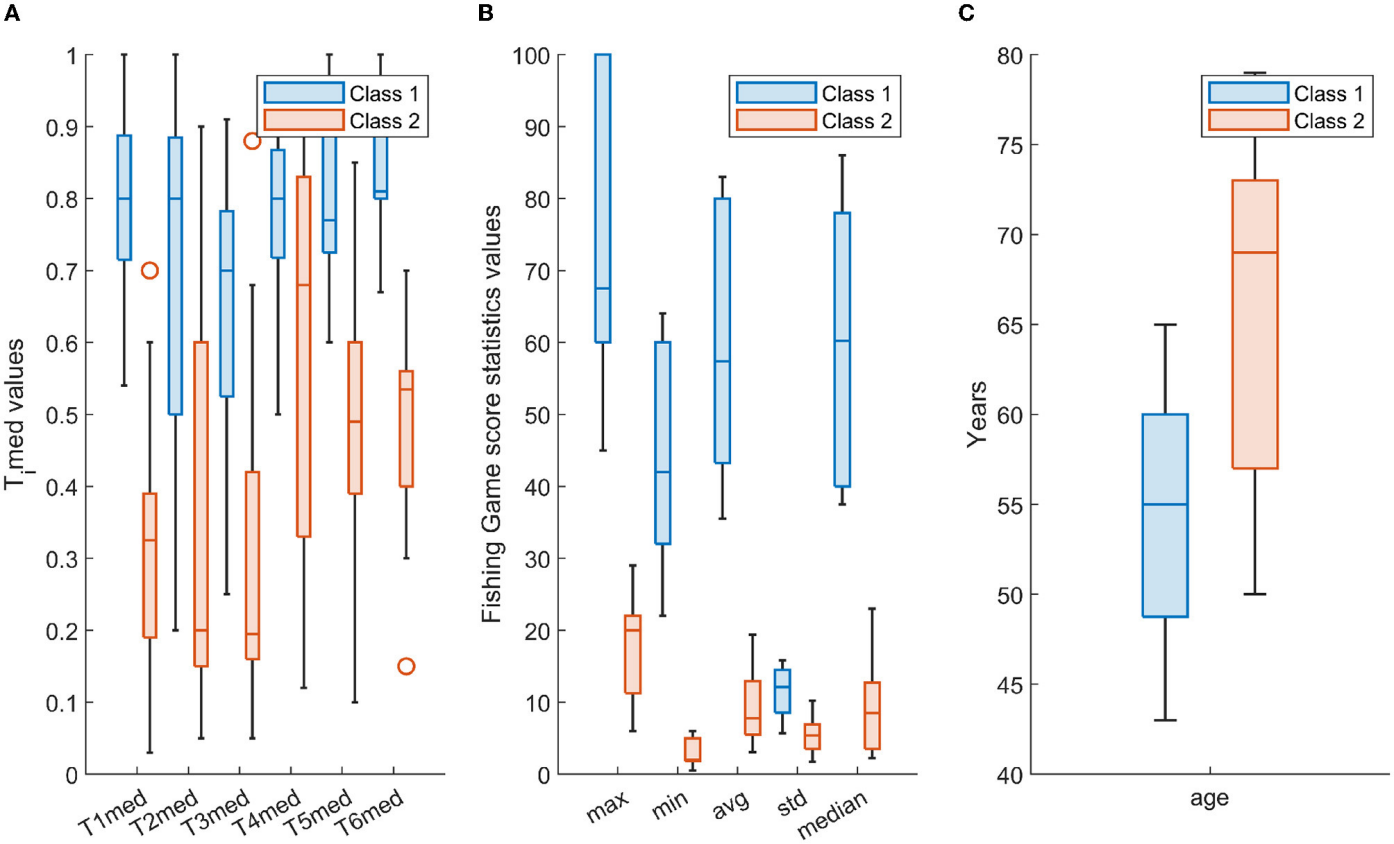
The distribution per class (class 1-cyan; class 2-orange) of **(A)** *Timed, i* = 1, 2, …, 6, **(B)** statistical parameters of the “Fishing Game” score, and **(C)** age, in the form of box-plotting. Each box ranges from the first quartile (Q1) to the third quartile (Q3) of the distribution and the range represents the interquartile range (IQR); the median is indicated by a line across the box and the “whiskers” on box plots extend from Q1 and Q3 to the most extreme data points; outliers are indicated with circles.

Figure 5 depicts the distribution of the employed features in the form of boxplots. In particular, for the two classes, i.e., class 1 and class 2, Figure 5A shows the boxplots of the *Timed, i* = 1, 2, …, 6, Figure 5B depicts the boxplots of the statistical parameters of the “Fishing Game” score, whereas Figure 5C illustrates the boxplots of the age parameter. As it can be seen from Figure 5, there are distinct differences between the two classes in many of the selected features; however, their combination, as reflected in the constructed scenarios, provides a robust representation of the two classes, explored by the adopted machine learning scheme (Figure 4).

#### 3.2.2. Employed Machine Learning

The aforementioned selected FVs were fed to two machine learning systems to perform the PD severity classification. The K-Nearest Neighbors (KNN) (Altman, 1992), Support Vector Machine (SVM) (Cortes and Vapnik, 1995), and Random Forest (RF) (Shi and Horvath, 2006) machine learning methods were adopted. KNN is mostly used in classification and regression problems, where existing nearby features are considered to be relatable. KNN estimates the similarity between two points by calculating the Euclidean distance between these adjacent features or points on a specific graph. SVM follows the concept of separating the features from one another, as the same types of features come on one plane, and another feature comes on another plane. The classifier is using a line for 1D, a plane for 2D, and hyperplanes for 3D data. The Ensemble classification learning method RF, which is also known as Random Decision Forests, relies during training on building stacks of decision trees. The final decision of the classification problem using RF classifiers is based on taking the average of all the decision trees outcomes, improving by that the accuracy of the prediction. In order to accommodate for the size of the analyzed dataset and avoid over-fitting, a Leave-One-Out Cross-Validation (LOOCV) method was adopted. In fact, LOOCV excludes one observation out of the training set of the dataset and performs validation only on that left out observation. This process is repeated as many as the number of observations included in the training set, computing by that the overall accuracy of the classifications of those left out observations to evaluate the performance of the classifier.

#### 3.2.3. Classification Performance Metrics

For each classifier (KNN, SVM, RF) and different FVs (1-6), the overall classification accuracy from the LOOCV scheme is computed, along with the metrics of recall and precision, all included in a confusion matrix, and F1 score along with Matthew's Correlation Coefficient (MCC), tabulated in Table 1. MCC is also included here, as it does not have the undesired characteristics of F1 score, i.e., not being normalized and not being symmetric (when swapping positive and negative cases), and is considered as a balanced measure, even when the classes are of very different sizes (Boughorbel et al., 2017). MCC returns a value ∈[−1, 1], with MCC=1 representing a perfect prediction, MCC=0 shows no better than random prediction and MCC=-1 indicates a total disagreement between prediction and observation.

**Table 1:** Classification performance metrics used in the study.

**Evaluation metric**	**Definition**	**Calculation**
Accuracy	Evaluates the model's percentage of correct predictions	$\frac{T_{P}+T_{N}}{T_{P}+T_{N}+F_{P}+F_{N}}$
Recall	Analyzes delays as true positive	$\frac{T_{P}}{T_{P}+F_{N}}$
Precision	Identifies true positive in the ground truth	$\frac{T_{P}}{T_{P}+F_{P}}$
F1 score	Analyzes the equilibrium between precision and recall	$\frac{2\times Precision\times Recall}{Precision+Recall}$
MCC	A correlation coefficient between the observed and predicted binary classifications	$\frac{T_{P}\times T_{N}-F_{P}\times F_{N}}{\sqrt{\left(T_{P}+F_{P}\right)\left(T_{P}+F_{N}\right)\left(T_{N}+F_{P}\right)\left(T_{N}+F_{N}\right)}}$

*T_P_, T_N_, F_P_, F_N_ denote the true positive, true negative, false positive, and false negative, respectively. MCC: Matthew's Correlation Coefficient*.

## 4. Results and Discussion

### 4.1. Demographics Impact

To study the impact of patients' demographics, i.e., age and gender, on the corresponding FVs (i.e., 2, 4, and 6) across the two classes (H&Y stage 1 and stage 2), a linear regression analysis was initially performed. Table 2 lists the estimated statistical significance level (*p*-value) for age and gender with the features related to the PGS and iMAT; boldface values denote statistically significant difference, i.e., *p* < 0.05. From the results presented in Table 2, age impacts only some of the iMAT score-related features, such as T1med, T3med, T5med, and T6med, and has no impact on the PGS score-related features. Moreover, gender shows no significant impact on all features; i.e., both from the PGS and iMAT.

**Table 2:** Estimated *p*-values from the demographics linear regression analysis.

	**PGS score-related features**					**iMAT score-related features**					
	**Avg**	**Med**	**Std**	**Min**	**Max**	**T1med**	**T2med**	**T3med**	**T4med**	**T5med**	**T6med**
Age	0.66	0.703	0.79	0.26	0.97	**0.001**	0.075	**0.018**	0.23	**0.012**	**0.0007**
Gender	0.56	0.49	0.99	0.79	0.84	0.88	0.55	0.56	0.66	0.19	0.64

*Statistically significance level: p < 0.05, denoted with boldface p-values*.

### 4.2. FVs Update

Based on the findings of Table 2, the initial versions of the FVs considered (1–6) are updated to the ones tabulated in Table 3, creating five scenarios for the classification process. In this way, the impact of age is only considered in the construction of the different FVs that include impactful demographics.

**Table 3:** The selected features per each feature vector (FV) scenario used as input to the K-Nearest Neighbors (KNN), Support Vector Machine (SVM), and Random Forest (RF) classifiers.

**FV scenarios**	**Selected features**
Scenario 1 (*N*=27)	{*T*_*i*_*med*|*i*∈(1, .., 6)}
Scenario 2 (*N*=27)	{*age, T*_*i*_*med*|*i*∈(1, .., 6)}
Scenario 3 (*N*=21)	{*jFishing*_*score*_|*j*∈(*max, min, avg, std, median*)}
Scenario 4 (*N*=21)	{*T*_*i*_*med, jFishing*_*score*_|*i*∈(1, .., 6)&*j*∈(*max, min, avg, std, median*)}
Scenario 5 (*N*=21)	{*age, T*_*i*_*med, jFishing*_*score*_|*i*∈(1, .., 6)&*j*∈(*max, min, avg, std, median*)}

*N denotes the number of patients with PD included in each FV scenario*.

### 4.3. Classification Results

**Figure 6:**
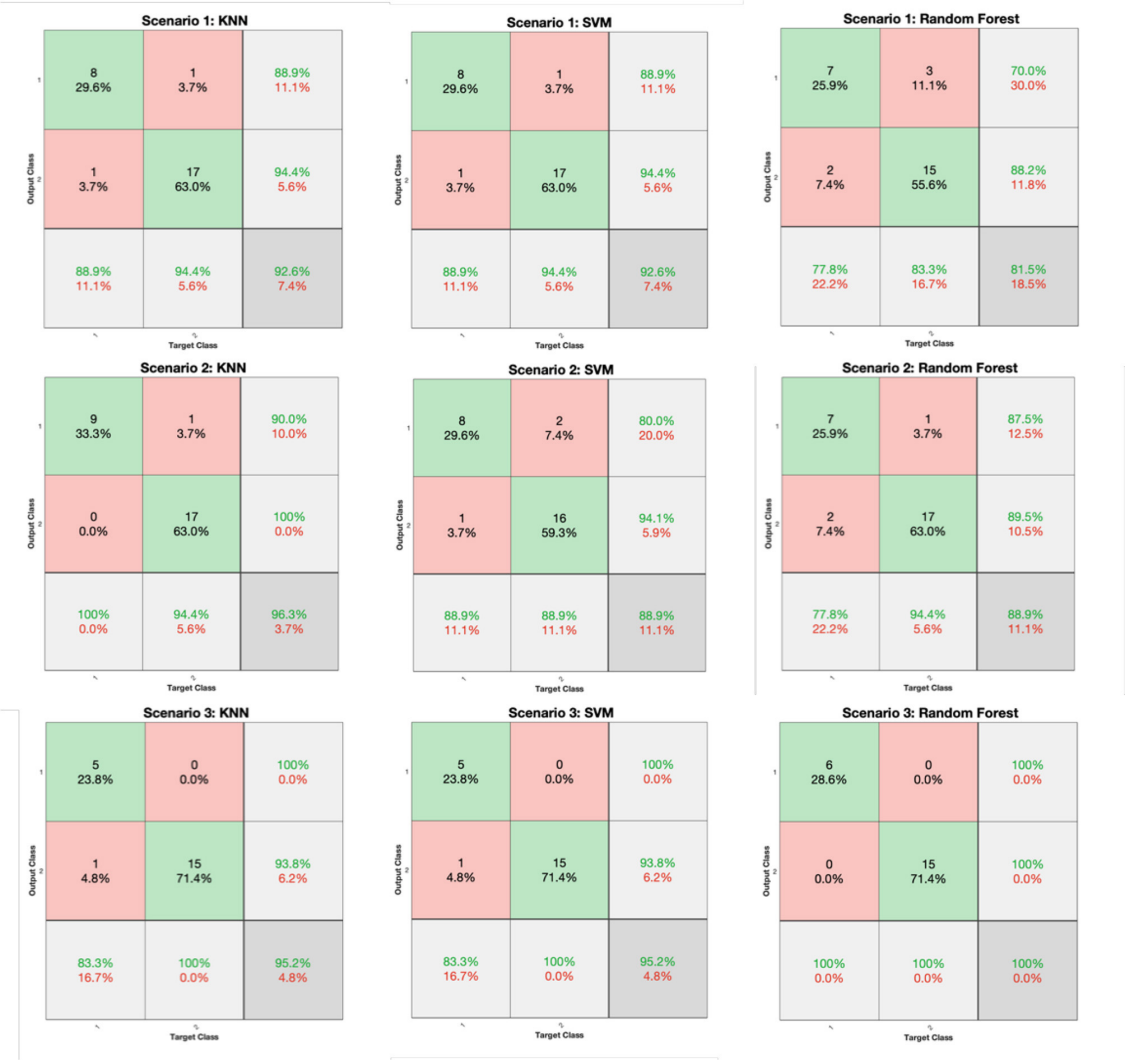
Confusion matrices generated from the K-Nearest Neighbors (KNN), Support Vector Machine (SVM), and RF classifiers for the feature vector (FV) scenarios of 1, 2, and 3 (Table 3).

**Figure 7:**
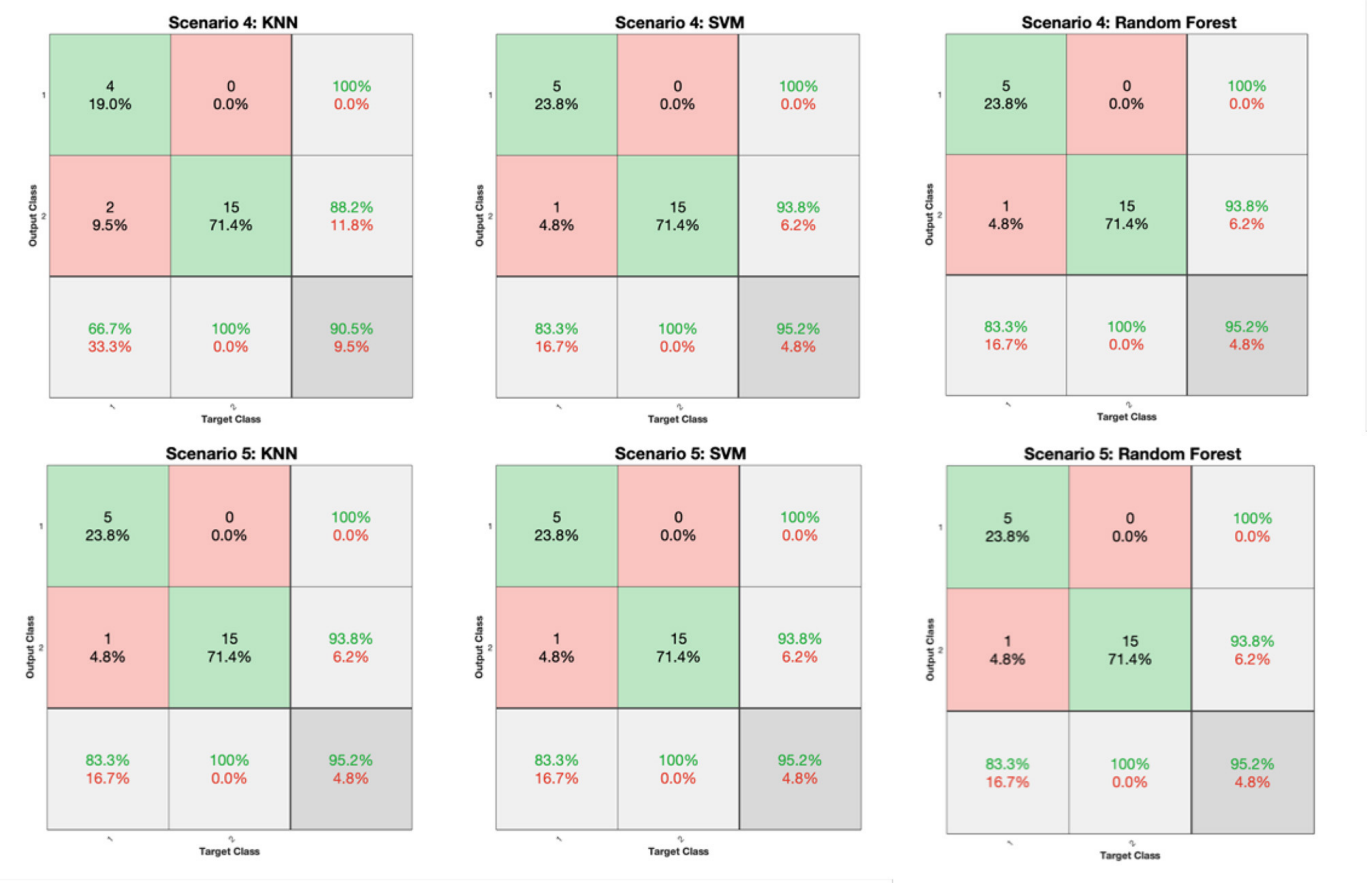
Confusion matrices generated from the KNN, SVM, and RF classifiers for the FV scenarios of 4 and 5 (Table 3).

The classification performance for all scenarios of Table 3 and for the KNN, SVM, and RF classifiers is depicted in Figure 6 (FV scenarios 1-3) and Figure 7 (FV scenarios 4, 5), respectively. Both figures present the related confusion matrices, which include the accuracy, precision, and recall metrics (Table 1), accordingly. Moreover, Table 4 tabulates the accuracy for each FV scenario (Table 3), combined with the corresponding F1 score and the MCC (Table 1).

**Table 4:** Leave-One-Out Cross-Validation (LOOCV) accuracy, F1 score, and MCC values achieved by all the scenarios using KNN, SVM, and RF classifiers.

	**Weighted KNN**					**Linear SVM**					**RF**				
	**Sc1**	**Sc2**	**Sc3**	**Sc4**	**Sc5**	**Sc1**	**Sc2**	**Sc3**	**Sc4**	**Sc5**	**Sc1**	**Sc2**	**Sc3**	**Sc4**	**Sc5**
Accuracy	0.926	0.963	0.952	0.905	0.952	0.926	0.889	0.952	0.952	0.952	0.815	0.889	1	0.952	0.952
F1 score	0.889	0.947	0.909	0.800	0.909	0.889	0.842	0.909	0.909	0.909	0.737	0.824	1	0.909	0.909
MCC	0.833	0.922	0.884	0.767	0.884	0.833	0.759	0.884	0.884	0.884	0.596	0.745	1	0.884	0.884

*Sc1-Sc5 corresponds to the FV Scenario 1-Scenario 5 of Table 3, respectively*.

Based on the results reported in Figures 6, 7, and Table 4, it is clear that in most cases, the information that is provided by the PGS/iMAT scores and their FV results has quite a satisfactory classification performance, in terms of the resulted metrics. More specifically, the FV scenario 1, which is solely based on the iMAT-related features, achieves a classification accuracy that ranges from 81.5% (RF) up to 92.6% (KNN, SVM). This performance is improved when the iMAT-related FVs are combined with the age information (FV scenario 2), extending the accuracy range from 88.9% (RF, SVM) up to 96.3% (KNN). When the PGS-related FVs are solely employed (FV scenario 3), the classification accuracy is increased in all classifiers' output, ranging from 95.2% (KNN, SVM) up to 100% (RF). When there is a combination of the PGS/iMAT-related FVs (FV scenario 4), the classification accuracy is still sustained high, ranging from 90.5% (KNN) up to 95.2% (SVM, RF). Finally, when the age is also combined (FV scenario 5), all classifiers converge to a 95.2% accuracy.

Considering the performance of the there classifiers, KNN sustains values greater than 90% accuracy across all FV scenarios, whereas SVM and RF have a wider range of performance, depending on the different FV scenarios, ranging from 88.9 up to 95.2% and from 81.5 up to 100%, respectively. This performance is also reflected in the corresponding values of F1 score/MCC (Table 4), with the RF exhibiting the lowest (0.737/0.596; FV scenario 1) and the highest (1/1; FV scenario 3) estimated values across the three classifiers. These results indicate that the least number of features (five in FV scenario 3) have produced the best performance in the case of RF, showing an ability to better capture the difference between the two classes, when compared to other FV scenarios (intra-comparison) and classifiers (inter-comparison). More stable performance is achieved by KNN, followed by the SVM, achieving an increased classification accuracy, as the FV is enriched with the two sources of information, i.e., PGS and iMAT scores, combined with the age. This showcases the potentiality of not only separately using PGS or iMAT to identify the current PD effect in a patient's motor skills but using them in a combinatory way as well.

Regarding the nature of the PGS and iMAT characteristics, it is evident that each of them provides useful information, captured in different settings. In fact, during the “Fishing Game,” the patient uses their trunk to control the game (Figure 2) and follows the flow of the game, which differs across the levels of the game and its sessions. Hence, it could be considered a task with variant content and unpredictability (e.g., game surprises, game challenges), in terms of motor interaction that the game demands from the user. This allows for more spontaneous, uncontrolled movements by the patient with PD. iMAT, however, requires the patient to follow specific movements that are performed by the expert (Figure 3), controlling as much as possible their movement, according to the specific test protocols. Hence, the related score expresses the patients' motor skills in controlled, less spontaneous, movements.

Clearly, the combination of both information sources, as seen by the employed classifiers, reveals their importance to arrive at an efficient classification of the stage of patients with PD (early or more advanced). This is further supported by the fact that the PGS/iMAT were designed following a co-creation approach, trying to gamify clinical assessment processes and quantify the PD symptoms in a more intuitive and naturalistic way. Hence, patients from their premises can have an intermediate use (e.g., every 10–15 days) of iMAT between periodic sessions of PGSs (e.g., daily or every 2 days per week), providing data that the proposed classification pipeline would use to infer for the improvement/deterioration of patients' PD motor skills in a longitudinal fashion. In this way, constructive feedback could be provided, both to the patient and his/her attending doctor, guiding more personalized and targeted interventions.

The proposed study continues the study presented in Dias et al. (2020b) and showcases the potentialities of the PGS and iMAT to efficiently monitor the motor skill status of patients with PD. Moreover, our results extend the work of Grammatikopoulou et al. (2019), where they analyzed the movement patterns of PD patients with early (6 patients) and advanced (12 patients) PD symptoms during playing a body motion-based video game. Their effort was to detect statistically significant differences between groups of different motor impairment levels based on their game performance. Their analysis resulted in statistically significant differences focused on the game duration, rather the game score *per se*, and body skeleton-based classification using deep learning that resulted in an accuracy of 77.7% between the two patient groups. Apparently, the results presented here showcase that the “Fishing Game” and iMAT scores provide better opportunities to evaluate the severity of PD and its effect on the motor skills of patients with PD. This is in line with the findings of the systematic review of Gallou-Guyot et al. (2022), who found that home-based active video games seem feasible, enjoyable, and safe and could be effective for the improvement of gait and balance functionality of patients with PD, being, at the same time, comparable to the usual care and conventional therapy.

The combinatory approach of PGS/iMAT suggested here could be used to connect the predominantly subjective evaluation in the clinical assessment of the PD patients' motor skills with a more enhanced technology-driven evaluation scheme. The latter could provide accurate indications about the disease staging, assisting the selection of personalized therapeutic interventions. As PD has a long prodromal phase, the PGS/iMAT could also be employed as an early PD stage monitoring tool, promoting physical activity and motor behavioral changes *via* a personalized gaming experience and different types of assessment.

### 4.4. Limitations and Future Work

Apparently, the limited number of patients with PD involved in the study constrains the generalization power of the findings. However, the latter is supported by the controlled environment used for data capturing and the clinical validation processes that were involved. Hence, the proposed study potentiates the extension of the current analysis to a larger scale of cohorts of patients with PD. This would also support the collection of big data from patients with PD at various disease severity stages, extending the binary classification problem examined here to a multi-class one. In addition, data acquired from the same patient for a long period of time could give rise to further validation and generalization of the classifiers' performance to inter/intra-subjects variability.

The study presented here examines one ExerGame from the PGS (“Fishing Game,”) also combined with the iMAT. However, PGS includes more ExerGames and additional categories of SGs (Figure 1). Further analysis will include data from pilots with an increased variety of PD patients' interaction with the PGS for a long (>6 months) period of time. This would allow the examination of the effect of the ExerGame characteristics on better revealing the motor skill status of patients with PD across time. Moreover, the extension of the iMAT to include fine motor skills assessment (e.g., handwriting testing) and/or additional assessment test that would target non-motor symptoms, such as voice degradation, emotional distress, unhealthy nutrition, low sleep quality, could lead to a holistic PD assessment tool. This could be combined with the whole range of SGs included in the PGS, enriching the information from integrated sources. Apparently, in that case, the adopted machine learning would be extended to deep learning classification schemes, that could create FV in the embedding space for efficient representation of the health status of patients with PD (Jiang et al., 2021). Finally, the transfer of the PGS/iMAT to an immersive (e.g., virtual reality) environment is also foreseen, in an effort to evaluate the level of user engagement and performance under more experiential interaction settings.

## 5. Conclusion

A machine learning-based approach for the estimation of the PD severity using features from the scores produced by the PD patients' interaction with PGS/iMAT was presented here. Data from 27 patients with PD at Stage 1 (9) and Stage 3 (18) from Greece, Germany, and the UK were used in the present study. Five feature vector scenarios, which either solely use the data from PGS or iMAT or data from their combination with the addition of the age, were explored for their efficiency to accurately represent the PD stage. Three machine learning classifiers (KNN, SVM, RF) were employed for the PD stage classification under an LOOCV scheme. The experimental results have shown that a high (>90%) classification accuracy of the PD stage is feasible from both data sources, i.e., PGS/iMAT. Clearly, these findings reinforce the role of the serious exergaming (and PGS, in general), along with the digital motor skill assessment, both combined in a unified NUI environment, to reflect the stage of patients with PD *via* machine learning. This perspective extends the horizon of the PD assessment to include more quantitative and objective means that could provide fine-grained metrics for remote symptoms' monitoring (such as motor skills degradation), shifting the locus of PD stage assessment from the clinic to the premise of the patient with PD.

## Data Availability Statement

The raw data supporting the conclusions of this article will be made available by the authors, without undue reservation.

## Ethics Statement

All data are drawn from the i-PROGNOSIS project and, especially the work of Dias et al. (2020b), in which all the experimental and ethical protocols were approved by the Bioethics Committee of the Aristotle University of Thessaloniki (AUTH) Medical School, Thessaloniki, Greece (401/31.01.2018), Ethik-Kommission an der Technischen Universität Dresden, Dresden, Germany (EK 451112017), and United Kingdom London—Surrey Borders Research Ethics Committee (18/LO/0074). Written informed consent was obtained from all patients with PD who participated in the study, for the publication of any potentially identifiable images or data included in this article.

## Author Contributions

DM and LH conceived, designed the whole analysis approach, and drafted the manuscript. SD provided the data within the PGS/iMAT conceptual framework. DM processed the data and performed the classification analysis. All the authors discussed the results, contributed to the finalization of the manuscript, and approved the submitted version.

## Funding

This research project was funded by the Abu Dhabi Department of Education and Knowledge (ADEK), UAE, under the Award for Research Excellence (AARE) 2018, ref. no: 29934 and partially supported by the Healthcare Engineering and Innovation Center (HEIC) fund from Khalifa University of Science and Technology.

## Conflict of Interest

The authors declare that the research was conducted in the absence of any commercial or financial relationships that could be construed as a potential conflict of interest.

## Publisher's Note

All claims expressed in this article are solely those of the authors and do not necessarily represent those of their affiliated organizations, or those of the publisher, the editors and the reviewers. Any product that may be evaluated in this article, or claim that may be made by its manufacturer, is not guaranteed or endorsed by the publisher.
